# Threat to Cefixime Treatment for Gonorrhea

**DOI:** 10.3201/eid1308.060948

**Published:** 2007-08

**Authors:** Shigeaki Yokoi, Takashi Deguchi, Tomomi Ozawa, Mitsuru Yasuda, Shin-ichi Ito, Yasuaki Kubota, Masayoshi Tamaki, Shin-ichi Maeda

**Affiliations:** * Gifu University, Gifu, Japan; † Toyota Memorial Hospital, Toyota, Japan

**Keywords:** *Neisseria gonorrhoeae*, cefixime, penicillin-binding protein 2, letter

**To the Editor:** From November 2002 through May 2003, 4 Japanese men, ranging in age from 23 to 45 years, visited the Department of Urology at Toyota Memorial Hospital, Toyota, Japan. Physical examinations showed urethral discharge and dysuria. Each had had sexual contact with sex workers in central Japan. Four strains of *Neisseria gonorrhoeae* were isolated from urethral specimens. Treatment comprised 200 mg cefixime, twice a day for 3 days. However, all 4 patients returned to the clinic with continuing symptoms, despite having completed the prescribed course of cefixime and abstaining from sexual activity. *N. gonorrhoeae* was again isolated from urethral swabs. Each patient was then treated with 1 g intravenous ceftriaxone. In the 3 patients who returned to the clinic for followup, the ceftriaxone treatment resulted in clinical and microbiologic cure.

Pulsed-field gel electrophoresis (PFGE) analysis of the *Spe*I-digested DNAs of *N. gonorrhoeae* was performed to assess the relatedness of pre- and posttreatment isolates (*1*). For these 8 isolates, MICs of penicillin G, tetracycline, cefixime, cefdinir, cefodizime, ceftriaxone, levofloxacin, azithromycin, and spectinomycin were determined on chocolate agar (GC) medium base supplemented with 1% IsoVital X (Becton Dickinson, Franklin Lakes, NJ, USA) and containing serial 2-fold dilutions of each agent (*2*). Media were inoculated with 10^4^ CFU and incubated at 35°C in 5% CO_2_ overnight. The MIC was defined as the lowest concentration inhibiting growth to <1 CFU. β-lactamase activity of the isolates was tested with a nitrocefin disk. The nucleotide sequences of the full-length *penA* gene encoding the penicillin-binding protein 2 (PBP 2) were identified in the isolates (*1*). Briefly, genomic DNAs from each isolate were subjected to PCR to amplify 3 fragments of the *penA* gene of *N. gonorrrhoeae*. PCR products were sequenced by the dye terminator method and with an automatic sequencer.

In each of the 4 cases, the PFGE patterns of the pre- and posttreatment isolates had the same numbers of bands (12–16 fragments), and the corresponding bands were the same apparent size; the pre- and posttreatment isolates were indistinguishable (*3*). MICs of antimicrobial agents for the 8 isolates are shown in the Table. All isolates were enzymatically negative for β-lactamase and possessed identical mosaic alterations in PBP 2. The mosaic PBP 2 was composed of fragments of PBP 2 from *N. cinerea* and *N. perflava* and was identical to that identified in our previous study (*1*).

**Table:** Antimicrobial drug susceptibilities of clinical isolates of *Neisseria gonorrhoeae* from patients with gonococcal urethritis treated unsuccessfully with a 3-day cefixime regimen*

Patient	MIC (μg/mL)									
	Isolate	PCG	TET	CFX	CFD	CDZ	CTX	LVF	AZM	SPC
1	Pre-Tx	4	2	0.5	1	0.125	0.125	16	0.25	16
	Post-Tx	4	2	0.5	1	0.125	0.125	16	0.125	32
2	Pre-Tx	8	4	0.5	1	0.25	0.5	8	0.5	32
	Post-Tx	8	2	0.5	1	0.125	0.25	8	0.5	16
3	Pre-Tx	4	2	1	2	0.25	0.125	16	0.25	16
	Post-Tx	4	2	1	2	0.25	0.125	16	0.25	16
4	Pre-Tx	4	1	1	2	0.25	0.25	16	0.125	16
	Post-Tx	4	2	1	2	0.25	0.25	16	0.25	32

*PCG, penicillin G; TET, tetracycline; CFX, cefixime; CFD, cefdinir; CDZ, cefodizime; CTX, ceftriaxone; LVF, levofloxacin; AZM, azithromycin; SPC, spectinomycin; Pre-Tx, isolate recovered before cefixime treatment; Post-Tx, isolate recovered after cefixime treatment, i.e., before ceftriaxone treatment.

Until recently, Japanese guidelines recommended oral administration of cefixime, 200 mg twice a day for 3 days to prolong the period of time for which the serum drug concentration remains above the MIC (*4*). However, treatment failure with this cefixime regimen was observed in our 4 cases of gonorrhea. The isolates showed cefixime MICs of 0.5–1 μg/mL and harbored mosaic alterations in PBP 2. Most recently, the emergence and spread of such strains in Japan (*1**,**5**,**6*) have led to the recommendation that ceftriaxone and spectinomycin should be used as the primary therapy for gonorrhea instead of oral cephalosporins (*7*).

In 2001, treatment failure was reported in a Caucasian man residing in Hawaii who had been given a single 400-mg dose of cefixime for gonorrhea (*8*). Pre- and posttreatment strains of *N. gonorrhoeae* were recovered from this patient, and 1 strain was isolated from his Japanese female sex partner who had visited Hawaii from Japan. Another pretreatment strain was isolated from a Micronesian man with gonorrhea residing in Hawaii, who had had sex with a woman from Malaysia or the Marshall Islands. This man was successfully treated with a single 400-mg dose of cefixime. For these strains, MICs of penicillin, tetracycline, spectinomycin, cefixime, ceftriaxone, ciprofloxacin, and azithromycin were 8.0, 4.0–8.0, <32, 0.25–0.5, 0.125, 8–16, and 0.125–0.25 μg/mL, respectively. The antibiograms of these strains in Hawaii were similar to those of strains with mosaic PBP 2 found in our 4 patients. The introduction to Hawaii of such multidrug resistant strains might be related to sex partners from Asia. Although the strains were not analyzed for alterations in PBP 2, they could have been derived from strains with cefixime resistance-associated mosaic PBP 2.

The strains with mosaic PBP 2 showed such decreased susceptibility to cefixime that they were not effectively eradicated by the 3-day treatment. Although it is not clear whether these strains are also resistant to the single 400-mg dose of cefixime (*8*, *9*), their emergence and spread could threaten treatment for gonorrhea with cefixime (*10*). Global emergence and spread of such multidrug-resistant strains of *N. gonorrhoeae* would be a matter of serious concern. The antimicrobial susceptibilities of current gonococcal isolates must be monitored periodically. In particular, posttreatment isolates from patients treated unsuccessfully with cefixime should be surveyed.
